# A voxel-based quantitative framework for analyzing the spatial redistribution and directionality of recurrence in glioblastoma

**DOI:** 10.1007/s11060-026-05471-0

**Published:** 2026-02-19

**Authors:** Takeshi Shimizu, Hirotaka Sato, Takahiro Sanada, Masato Saito, Nobuyuki Mitsui, Satoru Hiroshima, Manabu Kinoshita

**Affiliations:** 1 Department of Neurosurgery, Asahikawa Medical University, Midorigaoka-higashi 2-1-1-1, Asahikawa, Hokkaido 078-8510 Japan; 2 Department of Neurosurgery, The University of Osaka Graduate School of Medicine, Suita, Osaka Japan

**Keywords:** Glioblastoma, Recurrence, Tractography, Lesion mapping

## Abstract

**Introduction:**

Glioblastoma is an aggressive primary brain tumor that invariably recurs despite maximal resection and chemoradiotherapy. Understanding the spatial and directional dynamics of recurrence is crucial for informing treatment strategies, yet prior studies have relied mainly on qualitative classification schemes.

**Methods:**

We conducted a quantitative analysis of glioblastoma recurrence patterns using a two-pronged approach: voxel-based lesion mapping and vector analysis. Lesion distribution shifts between initial and recurrent tumors were statistically evaluated using anatomical labeling based on the AAL atlas. For directionality, we computed vectors from initial to recurrent lesion centroids and assessed their alignment with normative white matter fiber orientations derived from the Human Connectome Project.

**Results:**

Lesion mapping revealed a significant posterior shift of distribution in recurrence, particularly involving the parietal lobe. Vector analysis demonstrated that the recurrence vectors exhibited significant directional concordance with local white matter trajectories, as indicated by high mean absolute correlation coefficients (0.60 ± 0.23). These findings suggest that white matter pathways may guide tumor cell migration during recurrence.

**Conclusion:**

This study introduces a novel quantitative framework for assessing the spatial and directional features of glioblastoma recurrence. Our integrative analysis highlights the influence of structural brain connectivity on tumor spread and may ultimately contribute to refining initial treatment planning strategies.

**Supplementary Information:**

The online version contains supplementary material available at 10.1007/s11060-026-05471-0.

## Introduction

Glioblastoma is the most aggressive primary brain tumor in adults, characterized by inevitable recurrence despite maximal surgical resection of the contrast-enhancing lesion and standard chemoradiotherapy. This biological resilience has prompted efforts to explore more extensive initial treatment strategies [1, 2]. To successfully include radiographically occult regions in the primary treatment target, it is necessary to carefully balance oncologic rationale with preservation of essential brain functions. Knowledge of high-risk locations for future tumor recurrence could aid treatment planning for extended surgical resection or radiation therapy. Most prior studies on this subject have relied on qualitative or categorical descriptions, such as local versus distant or in-field versus out-of-field recurrence [3–7]. However, these classifications unfortunately fail to quantitatively capture the dynamics of glioblastoma’s progression over time, which is hypothesized to be driven along the white matter fiber tracts. This hypothesis has been supported by several qualitative assessments of recurrent glioblastoma lesions [8–10]. However, no attempt has been made to challenge this hypothesis to date from a quantitative perspective. A quantitative vector-based approach would enable more precise predictive modeling of recurrence patterns, thereby allowing the assessment of the directional relationship between recurrence and the underlying brain structures.

In this study, we aimed to elucidate the spatial redistribution of the lesion during recurrence in glioblastoma predominantly originating in the frontal lobe, focusing on directional alignment using a framework amenable to statistical testing. The objective of the study was to uncover potential anatomical constraints and migratory pathways that may guide glioblastoma recurrence, a finding that could ultimately help refine the theoretical basis for predicting glioblastoma’s recurrence locations.

## Materials and methods

### Patient cohort

Thirty consecutive patients with histologically confirmed newly diagnosed glioblastoma occurring in the frontal lobe were retrospectively enrolled in this study. Patients were treated between 2011 and 2023. Histological diagnoses were made according to the standard criteria at the time of initial diagnosis. For the purpose of the present analysis, tumor classification were retrospectively harmonized with the 2021 WHO classification of tumors of the central nervous system (CNS), 5th edition (WHO CNS 5), where applicable [11]. Because this retrospective cohort spans a long study period, including cases treated before routine molecular testing became standard, complete molecular information (e.g., IDH mutation status) was not available for all patients. Baseline clinical and pathological characteristics of the patients are summarized in Table S1. All included patients presented with the primary lesion located predominantly in the frontal lobe and underwent maximal safe resection, followed by standard chemoradiotherapy as appropriate, at Asahikawa Medical University Hospital. Tumor recurrence was radiologically confirmed in all cases. This study was approved by the institutional review board of Asahikawa Medical University (Approval No. 21041), confirming that all experiments will adhere to relevant guidelines and regulations and comply with the Declaration of Helsinki. Written informed consent was waived for this study due to its retrospective nature.

### Image acquisition and preprocessing

Gadolinium-enhanced T1-weighted images (GdT1) obtained at initial diagnosis and recurrence were used for tumor delineation and spatial normalization. The gadolinium-enhanced lesions were manually segmented in the native space, based on radiological consensus by two experienced neurosurgeons (TS and MK) (Figure S1). Non–contrast-enhancing T2-hyperintense regions, including peritumoral edema, were not included in the segmentation. The obtained voxels of interest (VOI) were further binarized and reviewed to ensure anatomical accuracy. Spatial normalization was performed by registering the original GdT1 to MNI152 using an affine transformation via the FMRIB Linear Image Registration Tool (FLIRT) with a mutual information algorithm with a 12-degrees-of-freedom transformation [12]. The derived transformation matrix was then applied to the corresponding VOI to generate a lesion mask on the standard MNI152 space. All registered masks were resliced to 1 mm isotropic resolution and stored in the Neuroimaging Informatics Technology Initiative (NifTI) format. To ensure hemispheric consistency, lesions in the left hemisphere were flipped to the right hemisphere. This hemispheric flipping was performed solely to standardize spatial orientation and to maximize statistical power for detecting common directional recurrence patterns at the group level, rather than to assess hemisphere-specific biological differences.

### Voxel-based lesion mapping

The lesions’ spatial distributions, both at initial and recurrent presentation, were performed by aggregating the VOIs across all patients on the standard MNI152 space [13]. Newly developed regions at tumor recurrence were identified by subtracting recurrent from the initial lesions for each subject in the standard MNI152 space, discarding voxels with negative values. The obtained voxels specific to tumor recurrence were then aggregated across all patients to generate a group-level recurrence map. All lesion maps were visualized as heatmaps using Volume Imaging in Neurological Research, Co-Registration and ROIs included (VINCI version 5.23.0) [14], allowing a quantitative voxel-wise regional frequency assessment of tumor occurrence across the entire cohort. Each VOI was annotated according to the Automated Anatomical Labeling atlas (AAL) [15]. The proportion of lesion voxels in each anatomical structure was calculated for both newly diagnosed and recurrent conditions. A chi-square test was performed to assess whether the spatial distribution of lesions significantly differed between the two timepoints, followed by Fisher’s exact test as a post hoc analysis.

### Directional vector analysis of tumor recurrence

**Fig. 1:**
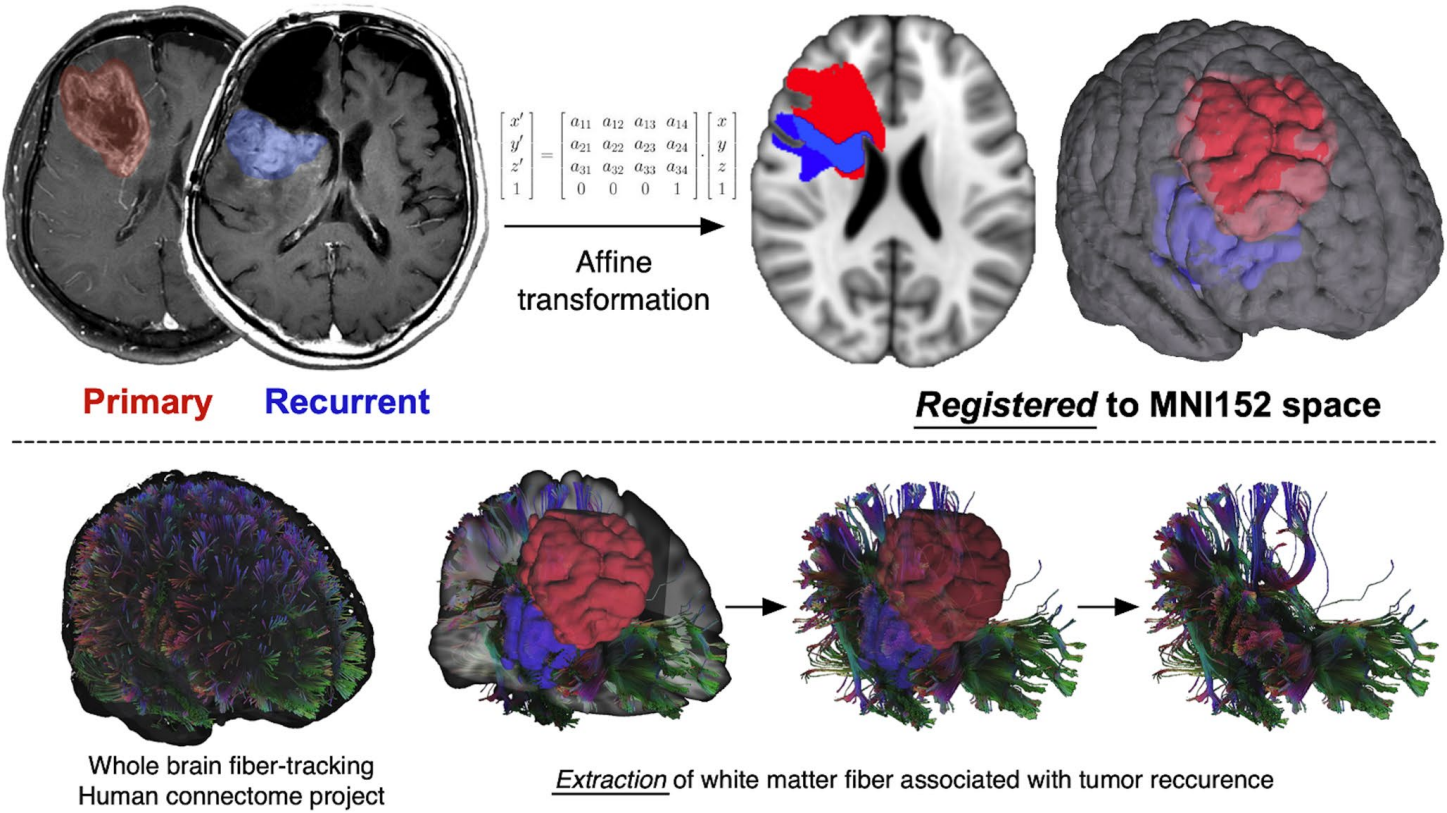
Tractography From Initial and Recurrent Glioblastoma Lesions. Representative tractography demonstrating fibers passing through both the initial and recurrent glioblastoma lesions in a single patient. Initial and recurrent contrast-enhancing lesions are used as regions of interest, and the connecting fiber pathways are visualized to illustrate potential anatomical routes of tumor progression

The vector analysis consisted of the following three computational steps, i.e., “Centroid vector calculation (the first step)”, “Fiber tracking and vector extraction (the second step)”, and “Correlation analysis (the final step)”. The overview of the workflow is presented in Fig. 1.

#### Centroid vector calculation

Centroids of the newly diagnosed and recurrent lesions were calculated in MNI152 space using FSL (fslstats -C) for each patient. A three-dimensional vector was then defined from the centroid of the newly diagnosed to the recurrent lesion, representing a spatial direction of tumor progression. The resulting vector was normalized to unit length, allowing for directional comparison downstream.

#### Fiber tracking and vector extraction

The current investigation opted to perform white matter fiber-tracking on healthy subjects and not on patients’ images. This approach not only allows analysis of cases lacking diffusion images but also avoids miscalculation during fiber tracking, which is affected by tumor presence and accompanying brain edema. Thus, diffusion data from the Human Connectome Project (HCP) were used as generalized q-sampling data for fiber tracking [16], and deterministic fiber tracking was performed using DSI Studio software (http://dsi-studio.labsolver.org) [17]. The VOIs corresponding to the newly diagnosed and the recurrent lesions were used as the seed and endpoint, respectively. Tracking parameters were as follows: Fractional Anisotropy threshold: 0.2, Step size: 0 mm, Minimum length: 1 mm, Maximum length: 25 mm, Fiber count: 5000. Fiber direction vectors were defined at the streamline level, with one representative direction vector assigned to each streamline, using custom post-processing of streamline data exported from DSI Studio. These vectors were normalized to unit length and used for subsequent directional similarity analyses.

#### Correlation analysis

**Fig. 2:**
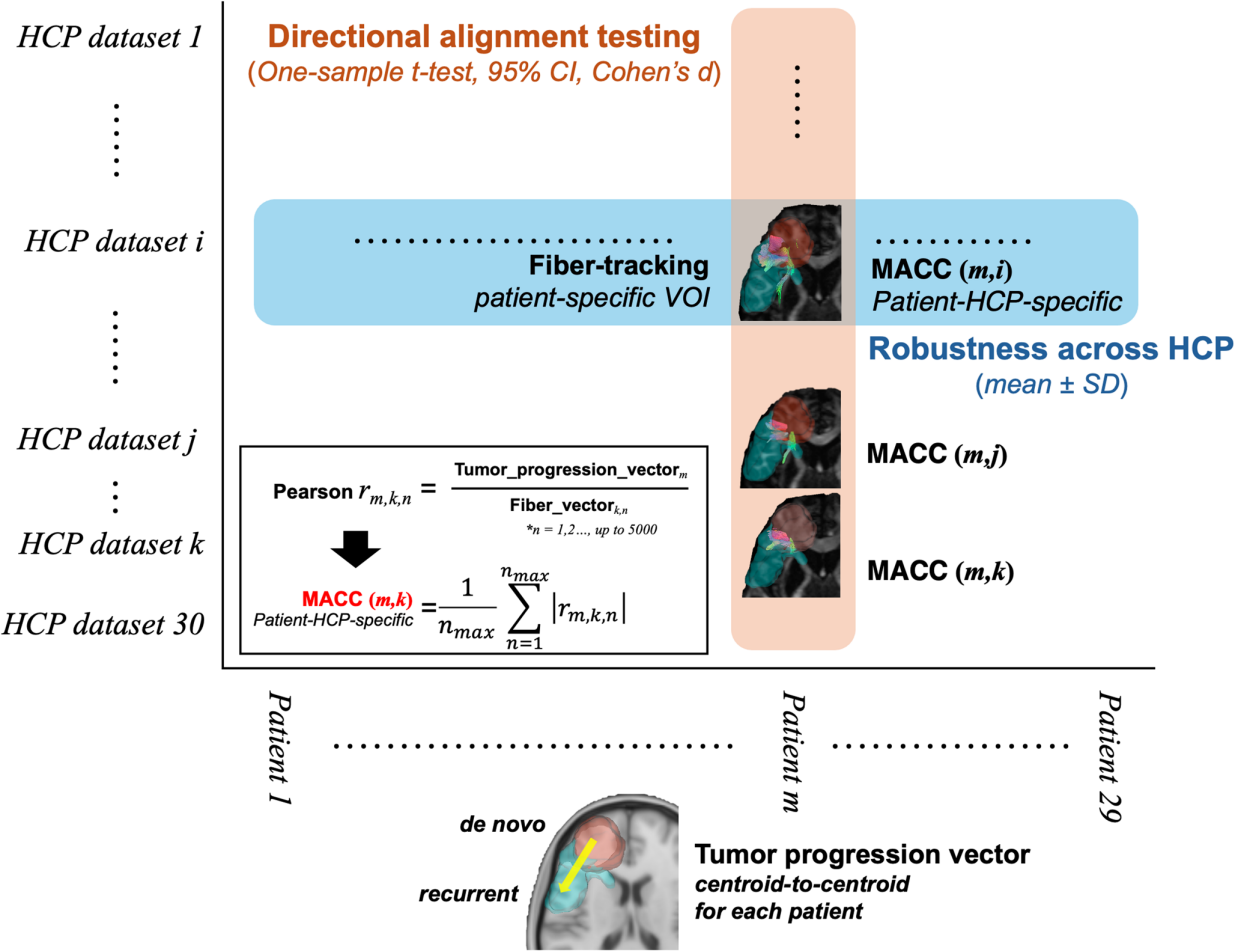
Conceptual Framework for MACC Aggregation and Robustness Assessment. Schematic illustration of the analytical framework used to quantify directional alignment between the tumor progression vector and local white matter fiber orientations (evaluated at the level of local fiber orientation vectors). The orange pathway represents aggregation of patient–HCP-specific MACCs across HCP-based diffusion subjects (*n* = 30) to derive a single patient-level MACC for each patient (*n* = 29), which is then used for cohort-level directional alignment testing. The blue pathway represents aggregation of patient–HCP-specific MACCs across patients to derive HCP-level MACCs, and variability across HCP subjects is used to assess robustness with respect to the choice of HCP-based diffusion data

The schematic representation of correlation analysis pipeline is presented in Fig. 2. For each patient, a directional vector representing tumor progression (the centroid-to-centroid vector from the newly diagnosed to recurrent lesion) and the corresponding set of local white matter fiber vectors were obtained as described above. To evaluate directional similarity, the Pearson product-moment correlation coefficient was calculated between the tumor progression vector and streamline-wise representative fiber direction vectors derived from tractography. The absolute values of the resulting correlation coefficients across all streamlines were averaged to yield a mean absolute correlation coefficient (MACC) for each patient. Because streamline vectors represent orientation rather than directional polarity, vectors pointing in opposite directions along the same axis were treated as equivalent, and the absolute value of the directional similarity metric was used. Correlation analysis was performed iteratively using diffusion data derived from 30 individuals randomly selected from HCP. This process was performed for 29 patients. One patient with distant recurrence was excluded from the analysis because a meaningful tumor progression vector and corresponding white matter fiber orientations could not be defined. Consequently, a total of 870 MACCs (29 patients x 30 HCP subjects) were obtained. To generate a single representative metric per patient, MACCs derived from the 30 individual HCP subjects were averaged, resulting in 29 patient-level MACC values. To assess the overall significance and consistency in tumor vector-white matter orientation across patients, a one-sample *t*-test was performed on patient-level MACC values (*n* = 29). The mean, standard deviation (SD), 95% confidence interval, *t*-statistic, and *p*-value were reported to evaluate whether the directional alignment was significantly different from chance. Effect size was calculated using Cohen’s d. A p-value < 0.05 was considered statistically significant. As a descriptive measure of robustness across HCP subjects, HCP-level mean and SD of MACC values were also calculated. As a supplementary robustness analysis, directional alignment was also evaluated using cosine similarity as an alternative sign-invariant metric (see Supplementary Methods).

## Results

### Voxel-based lesion mapping

**Fig. 3:**
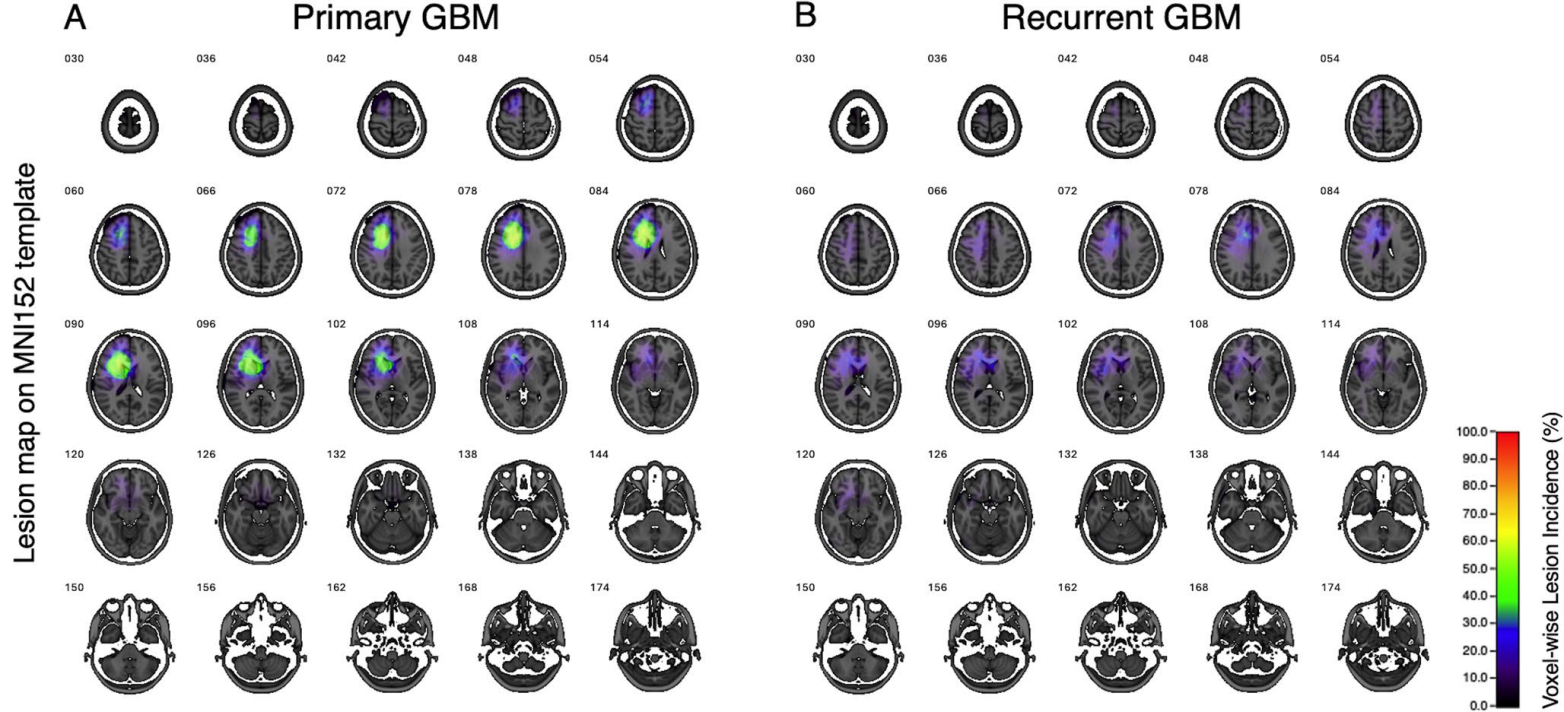
Voxel-Wise Lesion Mapping of Initial and Recurrent Glioblastoma. Voxel-wise lesion maps illustrating the anatomical distribution of contrast-enhancing glioblastoma at initial presentation (**A**) and at recurrence (**B**) after affine registration to MNI152 standard space. Color intensity represents the frequency of lesion overlap across patients (*n* = 30). Compared with initial lesions, recurrent lesions show a broader and more posterior distribution, including increased involvement of parietal regions

**Fig. 4:**
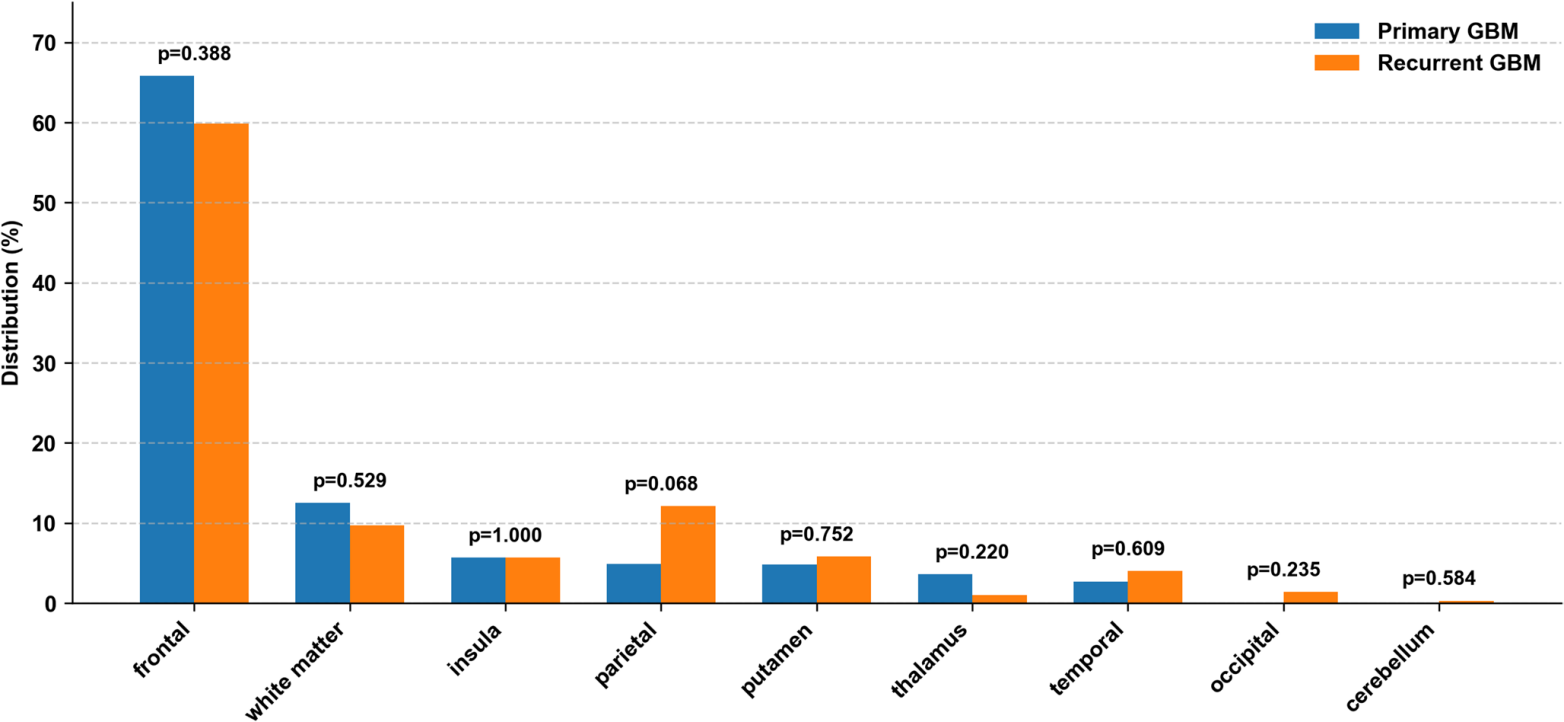
Regional Distribution of Primary and Recurrent Glioblastoma. Bar graph comparing the regional distribution of lesions between primary (blue) and recurrent (orange) glioblastoma. Values represent the relative proportion of the total lesion burden across regions. While primary tumors were predominantly localized in the frontal lobe, recurrent tumors demonstrated a posterior shift in distribution. Overall differences in regional distribution between primary and recurrent tumors were statistically significant (chi-square test, *p* < 0.001)

We first confirmed that the analyzed cohort consisted of glioblastomas with lesions strictly confined to the frontal lobe. Newly diagnosed lesions were most frequently located in the frontal as designed, but also extended into the temporal lobe (Fig. 3A). On the other hand, while the recurrent lesions mostly occurred at locations similar to those at the newly diagnosed setting, we observed a wider spread of lesions extending beyond the frontal lobe. They showed a broader distribution, extending more posteriorly, particularly into the parietal lobe (Fig. 3B). It was also noted that recurrent lesions did not accumulate in specific locations as did the newly diagnosed lesions. This was obvious by the absence of “hotspots” for the recurrent lesions (Fig. 3B). Quantitative measurement of the lesion location objectively confirmed this observation. Figure 4 shows the frequency of the lesion occupying each brain segment, referring to the MNI152 structural atlas. A chi-square test demonstrated a statistically significant difference in the spatial distribution of lesions between the newly diagnosed and recurrent lesions (*p* < 0.001). A post hoc analysis using Fisher’s exact test revealed a trend toward increased recurrence in the parietal lobe (*p* = 0.068), though this did not reach statistical significance (Fig. 4).

### Directional vector analysis of tumor recurrence

**Fig. 5:**
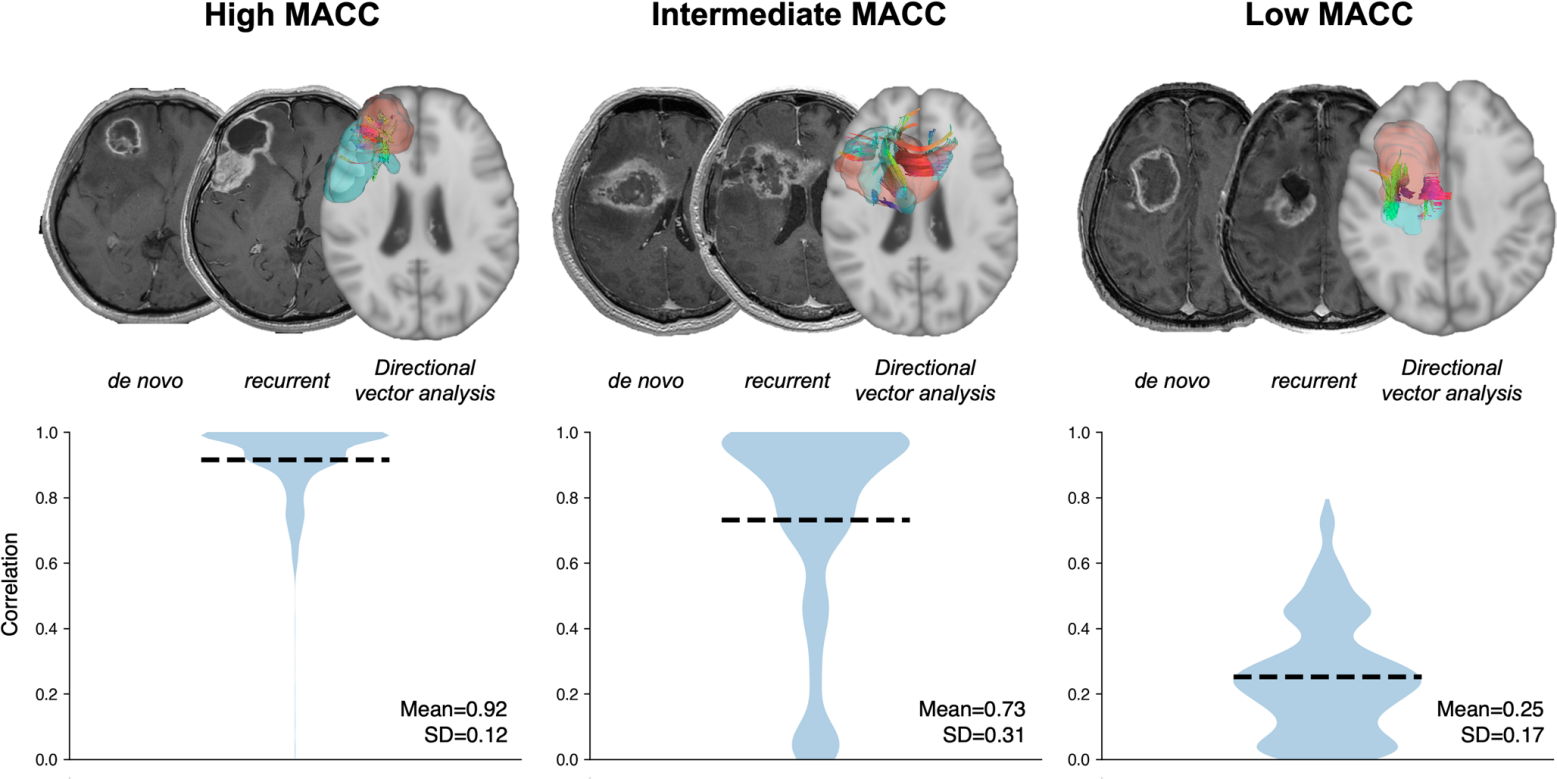
Representative examples of fiber-level directional concordance. Representative patients with high, intermediate, and low MACC values are shown. Upper panels depict diffusion MRI–based fiber tractography (axial view). Lower panels show fiber-level correlation coefficients between the tumor progression vector and individual fiber vectors. In intermediate- and low-MACC cases, both aligned and non-aligned fibers are present, resulting in increased variability and lower overall MACC values

Directional vector analysis was performed for 29 patients, excluding one due to a remote recurrence (Figure S2). Across the cohort, the mean of patient-level MACC between tumor progression and white matter fiber was 0.60, with a standard deviation of 0.23. A one-sample *t*-test confirmed that the directional alignment was significantly different from random chance (*t* = 13.9, *p* < 0.0001, 95% CI: 0.51–0.69). The effect size was large enough to indicate a robust directional association between tumor recurrence and white matter tract orientation (Cohen’s d = 2.58). The mean MACC at the HCP level averaged across patients was highly consistent across different HCP subjects (mean = 0.60, SD = 0.025; *n* = 30), indicating low sensitivity of patient-level MACC against the choice of HCP diffusion data. Figure 5 illustrates representative examples of fiber-level directional concordance in patients with high, intermediate, and low mean MACC. Fiber-level correlations showed relatively consistent directional concordance for a case with high mean MACC (Fig. 5A). However, cases with intermediate- to low-mean MACC exhibited greater variability (Fig. 5B, C), with both aligned and non-aligned fibers. Figure S3 is a correlation heatmap between vectors of patient-specific tumor progression and local white matter. Cross-correlation was performed between 29 patients and 30 randomly selected HCP subjects. Each cell in the matrix represents the mean MACC between a patient’s tumor vector and the white matter fiber tract orientation of a specific HCP subject. The heatmap illustrated an overall high level of directional correlation between patient-specific tumor progression and local white matter vectors. It should be noted that one case showing MACC = 0 is the case that exhibited remote recurrence.

## Discussion

While previous reports have primarily relied on qualitative or categorical descriptions of recurrence patterns [3–7], the current investigation aimed to quantitatively characterize the spatial dynamics of glioblastoma recurrence using voxel-based lesion mapping and directional vector analysis. We first employed voxel-based lesion mapping to compare the anatomical distribution of initial and recurrent tumors. We then introduced a novel vector analysis that evaluates the directional concordance between tumor progression vectors and normative white matter tractography. The findings highlighted that both the distribution and directionality of tumor recurrence can be rigorously assessed in a quantitative framework.

### Spatial redistribution during recurrence

Lesion mapping in this study revealed a marked spatial shift in recurrence patterns of glioblastoma. When initial tumors are predominantly located in the frontal lobe, recurrent lesions extend more broadly and posteriorly, with notable involvement of the parietal lobes. This transformation in lesion distribution was objectively quantified using anatomical labeling based on the AAL atlas [15] and statistically evaluated through chi-square and Fisher’s exact tests. This analytical approach enables not only the visualization of lesion topography but also the quantitative measurement of recurrence pattern, thereby converting what has traditionally been a descriptive analysis into a quantifiable framework. To enhance analytical precision and reduce the required sample size, the present study deliberately focused on patients with initial lesions confined to the frontal lobe. By incorporating spatial context into the evaluation of tumor dynamics, our findings underscore the highly adaptive and migratory nature of glioblastoma progression and provide important insights into its preferred patterns of recurrence.

### Glioblastoma spread directionality and white matter influence

In addition to regional redistribution, we further examined the directional tendency of tumor recurrence using a novel vector-based approach. The rationale for this analysis stems from the long-standing observation that glioblastoma often spreads not randomly, but along specific anatomical routes, many of which coincide with densely packed white matter structures (8–10). To quantitatively capture this behavior, we defined individual vectors connecting the centroids of initial and recurrent lesions for each patient, thereby encoding the spatial direction of tumor progression. These vectors were compared with the local orientation of white matter tracts derived from the HCP normative tractography subjects [16]. We used a normative connectome to represent population-level white matter architecture in a common reference space, enabling consistent estimation of local fiber orientation across subjects. Patient-specific diffusion tractography in glioblastoma can be unreliable due to tumor-related distortion, edema, and tractography’s known susceptibility to false-positive streamlines and limited handling of complex crossings; thus, a normative approach was chosen for robustness and comparability [18, 19]. Notably, these comparisons revealed a significant directional alignment between tumor progression vectors and the surrounding white matter architecture. The fiber-level variability observed in intermediate- and low-MACC cases suggests that a lower MACC does not indicate the absence of directional tumor spread but rather reflects heterogeneous engagement of surrounding white matter pathways. The consistency of this alignment, quantified as high MACC, was statistically robust, suggesting a pre-existing anatomical constraint for the spread of frontal glioblastoma, such as deep frontal-parietal or callosal tracts [20, 21].

Furthermore, the current study, to the best of our knowledge, is the first to quantitatively validate the directional relationship between glioblastoma and white matter fiber tracts. While previous studies have qualitatively noted the alignment of glioma spread with white matter tracts based on visual assessment, no such attempt that matches the current quantitative approach has been conducted. The proposed method reduces observer bias and provides a replicable and anatomically grounded framework. These findings suggest that white matter orientation may act as a latent scaffold influencing glioblastoma recurrence and highlight its potential utility in postoperative surveillance and radiotherapeutic planning. Recent studies have suggested that brain connectivity and white matter–derived metrics may carry prognostic relevance in glioblastoma, linking network-level features to tumor spread, recurrence patterns, and survival [22–24]. These observations motivate future investigations to examine whether directional alignment measures, such as the MACC proposed here, are associated with clinical outcomes including progression-free or overall survival.

### Clinical implications and future directions

The spatial and directional analytical framework presented in this study offers a novel strategy for anticipating recurrence patterns in glioblastoma. Notably, the directional concordance between tumor progression and white matter tracts highlights the potential to identify high-risk anatomical pathways for recurrence based on individual white matter architecture. This finding may have direct clinical implications. Incorporating recurrence directionality into postoperative imaging surveillance and radiotherapy planning could facilitate earlier detection and more precise targeting, thereby improving local disease control. Prior studies have suggested that early radiological changes on advanced MRI modalities may serve as indicators of imminent recurrence, even before conventional imaging becomes diagnostic [25]. Moreover, the preferential localization of recurrence has previously been analyzed in relation to radiotherapy dose distribution and anatomical vulnerability [26–28]. Our directional findings extend this knowledge by offering a quantitative, anatomy-based prediction model that may refine risk stratification for recurrence-prone regions.

### Limitations

Several limitations should be acknowledged. The cohort prepared for the current study was intentionally restricted to patients with frontal lobe-originating glioblastoma to reduce anatomical heterogeneity and enhance sensitivity for detecting consistent spatial and directional recurrence patterns. Consequently, the sample size inevitably turned relatively small, which may limit the generalizability of our findings. Future studies involving larger, multicenter cohorts are needed to validate the reproducibility of the spatial and directional trends observed in this study. Moreover, because lesions varied in size, shape, and precise location, spatial normalization and atlas-based anatomical labeling may introduce residual misalignment and uncertainty in ROI delineation. This trade-off is inherent to atlas-based voxelwise approaches and should be considered when interpreting fine-grained regional effects.

Although viable tumor components may exist beyond contrast-enhancing regions, precise differentiation of non–contrast-enhancing tumor from edema using conventional imaging remains challenging, and thus such regions were not included in the present analysis.

There is also potential uncertainty in deterministic tractography. While it offers intuitive visualization of white matter trajectories, it remains susceptible to false positives and cannot capture crossing fibers with high precision [29]. Advanced tractography methods, such as probabilistic or global approaches, may provide more robust assessments of white matter architecture.

In addition, we standardized hemispheric orientation by flipping left-hemisphere tumors to the right to improve group-level comparability; however, laterality itself may influence growth and recurrence patterns. Future studies with larger, hemisphere-balanced cohorts could explicitly examine hemisphere-dependent differences, including complementary reciprocal-flipping strategies as a stability analysis.

Finally, although our analysis demonstrated statistical associations between tumor recurrence vectors and white matter orientation, causal mechanisms remain speculative. It is possible that other microenvironmental factors, such as regional cell density, vascular patterns, or tumor–stroma interactions, could confound this relationship [30]. Further investigation combining radiographic, histopathological, and molecular features will be necessary to elucidate the biological underpinnings of these spatial dynamics. Future studies with larger cohorts may extend this framework to tract-specific analyses to determine whether glioblastoma recurrence exhibits preferential alignment with particular white matter pathways.

## Conclusion

This study provides a novel quantitative framework for evaluating the spatial and directional characteristics of glioblastoma recurrence. By combining lesion mapping with fiber-based vector analysis, we demonstrated that recurrent tumors not only shift their anatomical distribution but also exhibit a directional alignment with white matter pathways. These findings underscore the potential influence of structural connectivity on tumor spread and offer a new perspective for understanding and predicting glioblastoma progression, which may ultimately inform initial treatment strategies.

## Supplementary Information

Below is the link to the electronic supplementary material.

Supplementary Material 1: Supplementary table S1. Clinical characteristics and treatment summary of the study cohort. Baseline characteristics, treatment details, and outcomes of patients with newly diagnosed frontal lobe glioblastoma are shown. Extent of resection was assessed using the RANO classification. Values are presented as mean ± standard deviation unless otherwise indicated

Supplementary Material 2: Supplementary figure S1. Representative examples of contrast-enhancing tumor segmentation at initial diagnosis and recurrence. Representative gadolinium-enhanced T1-weighted images with manual segmentation masks are shown at initial diagnosis and at recurrence. Non–contrast-enhancing T2-hyperintense regions (including peritumoral edema) were excluded

Supplementary Material 3: Supplementary figure S2. Case of remote recurrence excluded from directional Analysis. The only case with spatially remote recurrence of glioblastoma relative to the initial lesion is presented. The recurrent lesion is anatomically disconnected from the primary tumor site, precluding meaningful definition of a tumor progression vector. Consistently, fiber tracking performed using diffusion data from all 30 HCP subjects yielded no connecting streamlines between the initial and recurrent lesions. This case was therefore excluded from the directional vector analysis

Supplementary Material 4: Supplementary figure S3. Heatmap of correlation between tumor progression vectors and HCP fiber Orientations. Heatmap showing MACCs between tumor progression vectors and the orientation of white matter fibers for each patient-HCP pairing. Rows correspond to individual HCP subjects (*n* = 30), and columns correspond to glioblastoma patients (*n* = 29). Warmer colors indicate stronger directional alignment between tumor progression and white matter orientation

Supplementary Material 5

## Data Availability

The datasets generated and/or analyzed during the current study are available from the corresponding author on reasonable request.

## Abbreviations

CNS: The central nervous system
GdT1: Gadolinium-enhanced T1-weighted images
VOI: Voxels of interest
FLIRT: FMRIB Linear Image Registration Tool
NifTI: Neuroimaging Informatics Technology Initiative
FSL: FMRIB Software Library
AAL: Automated Anatomical Labeling atlas
HCP: Human Connectome Project
MACC: Mean Absolute Correlation Coefficient
