# Supplementary material for: A voxel-based quantitative framework for analyzing the spatial redistribution and directionality of recurrence in glioblastoma
Source: J Neurooncol. 2026 Feb 19;177(1):8. doi: 10.1007/s11060-026-05471-0 (PMC12920414; doi:10.1007/s11060-026-05471-0)
Supplement: Supplementary file 1 — Supplementary Material 1: Supplementary table S1. Clinical characteristics and treatment summary of the study cohort. Baseline characteristics, treatment details, and outcomes of patients with newly diagnosed frontal lobe glioblastoma are shown. Extent of resection was assessed using the RANO classification. Values are presented as mean ± standard deviation unless otherwise indicated [file 11060_2026_5471_MOESM1_ESM.pdf]

Supplementary Table S1. Clinical characteristics and treatment summary

	All patients (n = 30)
**Demographics**	
Age, years	62.9
Sex, male, n (%)	19 (63)
Laterality (right), n (%)	19 (63)
**Tumor characteristics**	
IDH status	
Mutant, n (%)	—
Wild-type, n (%)	23 (77)
Not available, n (%)	7 (23)
**Treatment**	
Extent of resection (RANO*resect* classification)	
Class 1 (gross total), n (%)	5 (17)
Class 2A/2B (near total), n (%)	11 (37)
Class 3A/3B (subtotal), n (%)	11 (37)
Class 4 (biopsy), n (%)	3 (10)
Radiotherapy, n (%)	25 (83)
Concurrent TMZ, n (%)	23 (77)
Adjuvant TMZ, n (%)	21 (70)
**Clinical outcomes**	
Progression-free survival, days	254.4
Overall survival, days	510
Censored cases, n (%)	7 (23)
Median follow-up, days	1798
