# Supplementary figures and images for: A voxel-based quantitative framework for analyzing the spatial redistribution and directionality of recurrence in glioblastoma
Source: J Neurooncol. 2026 Feb 19;177(1):8. doi: 10.1007/s11060-026-05471-0 (PMC12920414; doi:10.1007/s11060-026-05471-0)

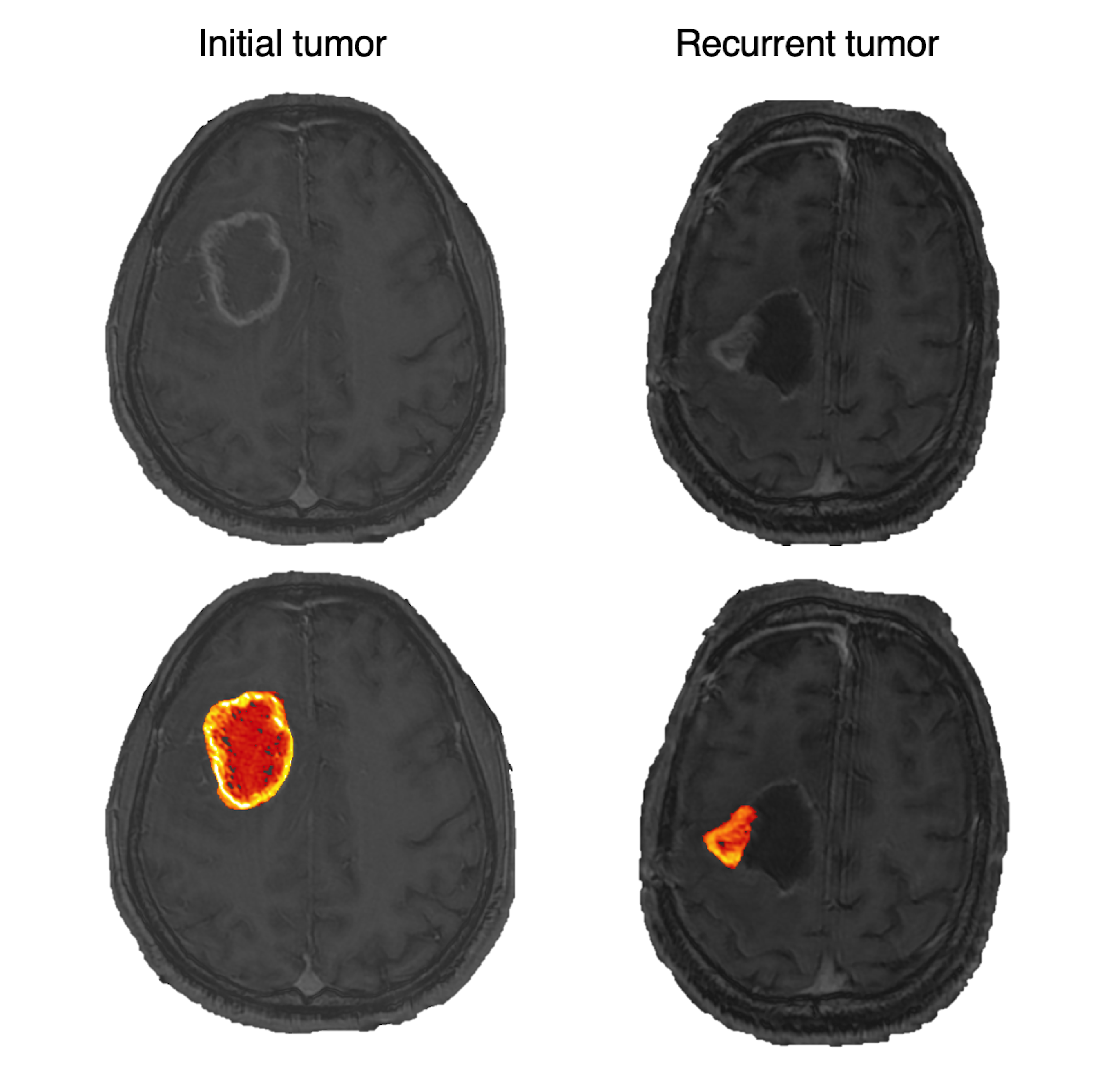

Supplement: Supplementary file 2 — Supplementary Material 2: Supplementary figure S1. Representative examples of contrast-enhancing tumor segmentation at initial diagnosis and recurrence. Representative gadolinium-enhanced T1-weighted images with manual segmentation masks are shown at initial diagnosis and at recurrence. Non–contrast-enhancing T2-hyperintense regions (including peritumoral edema) were excluded [file 11060_2026_5471_MOESM2_ESM.tiff]

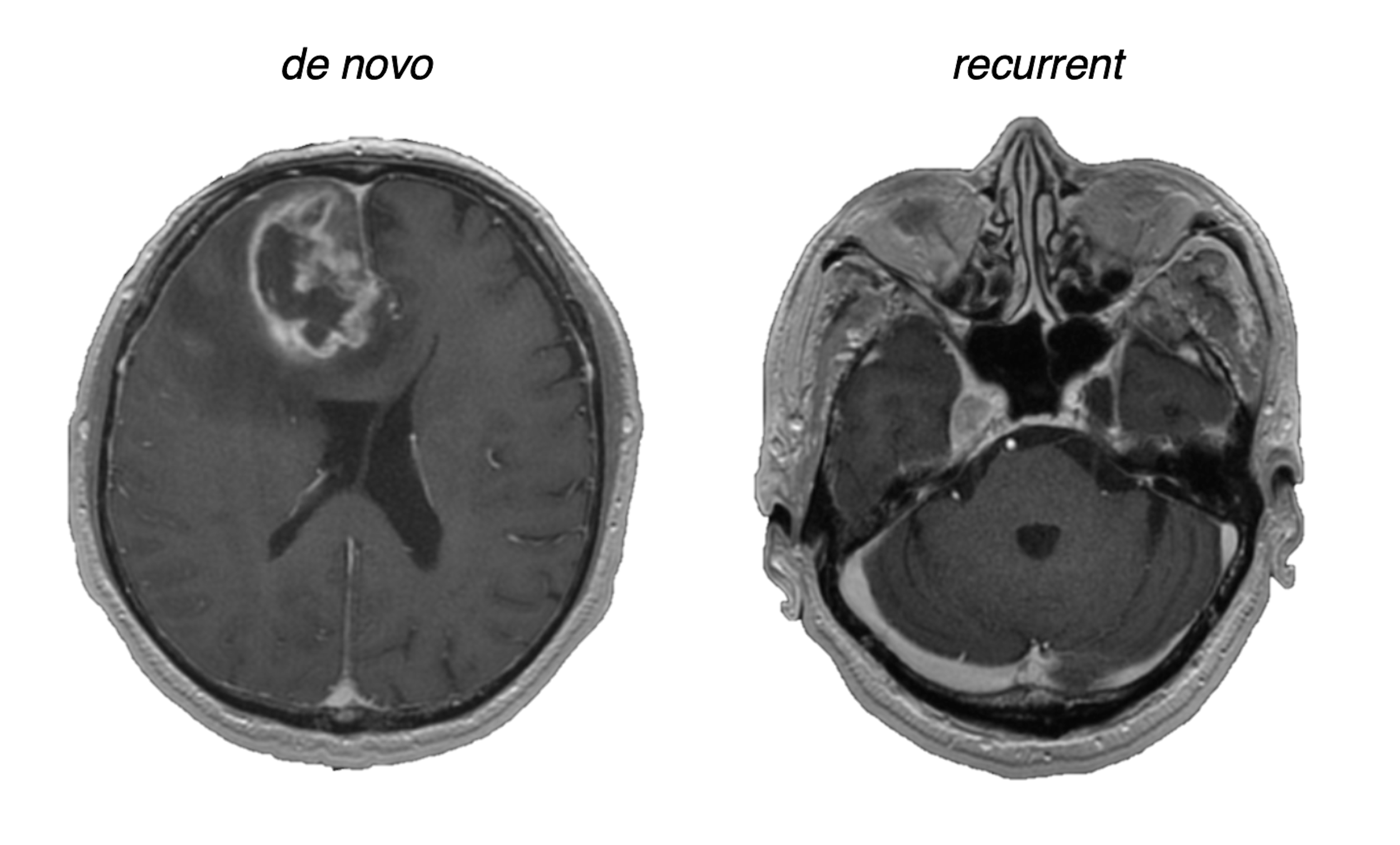

Supplement: Supplementary file 3 — Supplementary Material 3: Supplementary figure S2. Case of remote recurrence excluded from directional Analysis. The only case with spatially remote recurrence of glioblastoma relative to the initial lesion is presented. The recurrent lesion is anatomically disconnected from the primary tumor site, precluding meaningful definition of a tumor progression vector. Consistently, fiber tracking performed using diffusion data from all 30 HCP subjects yielded no connecting streamlines between the initial and recurrent lesions. This case was therefore excluded from the directional vector analysis [file 11060_2026_5471_MOESM3_ESM.tiff]

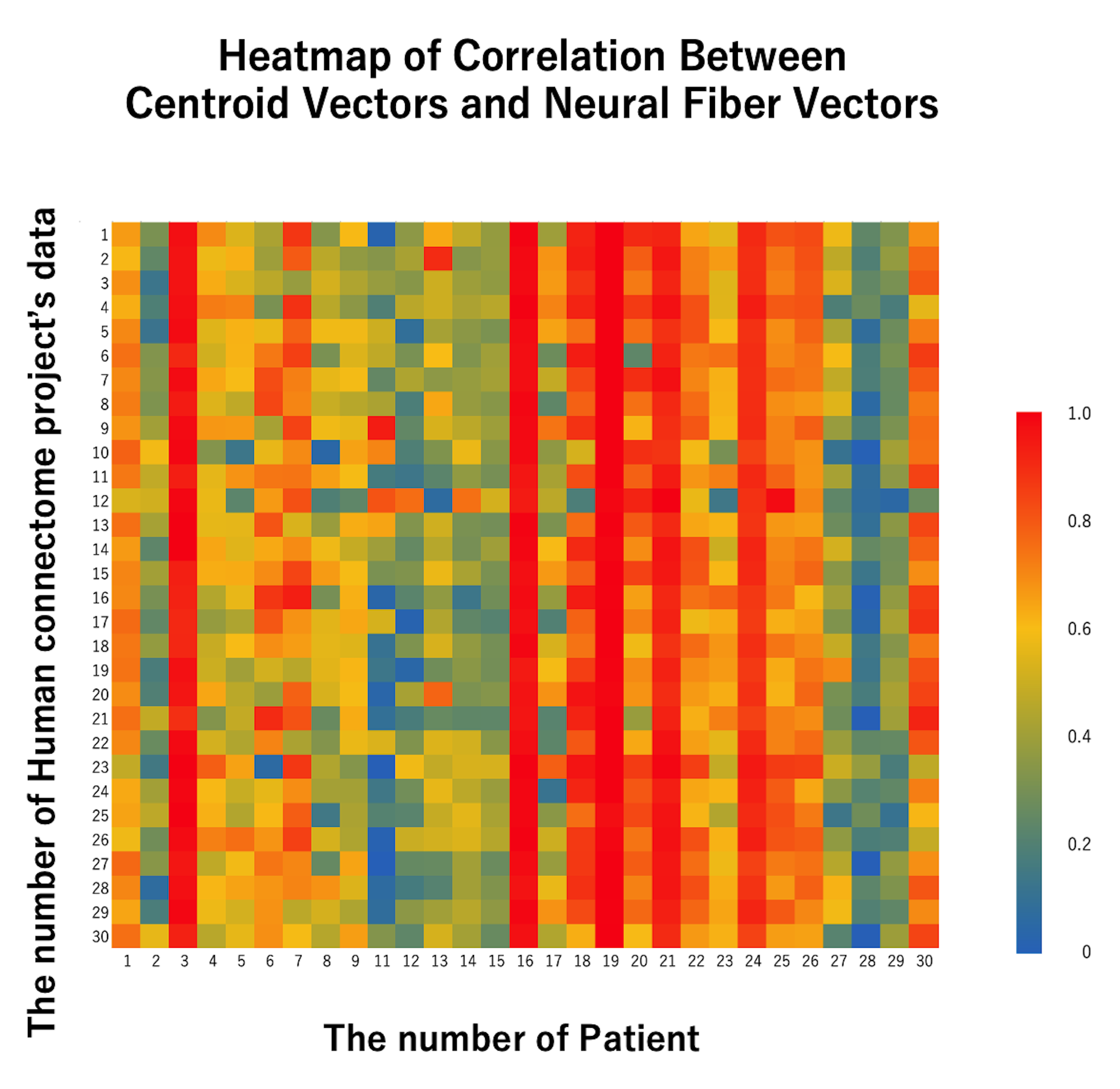

Supplement: Supplementary file 4 — Supplementary Material 4: Supplementary figure S3. Heatmap of correlation between tumor progression vectors and HCP fiber Orientations. Heatmap showing MACCs between tumor progression vectors and the orientation of white matter fibers for each patient-HCP pairing. Rows correspond to individual HCP subjects (n = 30), and columns correspond to glioblastoma patients (n = 29). Warmer colors indicate stronger directional alignment between tumor progression and white matter orientation [file 11060_2026_5471_MOESM4_ESM.tiff]
