# Supplementary material for: A voxel-based quantitative framework for analyzing the spatial redistribution and directionality of recurrence in glioblastoma
Source: J Neurooncol. 2026 Feb 19;177(1):8. doi: 10.1007/s11060-026-05471-0 (PMC12920414; doi:10.1007/s11060-026-05471-0)
Supplement: Supplementary file 5 — Supplementary Material 5 [file 11060_2026_5471_MOESM5_ESM.pdf]

Cosine Similarity Analysis

Methods:

As a robustness analysis, cosine similarity was used as an alternative sign-invariant metric to quantify directional alignment between tumor progression vectors and local white matter fiber orientation vectors.

Tumor progression vectors and local fiber orientation vectors were normalized to unit length, and cosine similarity was computed as their dot product. Absolute cosine similarity values were averaged across all local fiber orientation vectors for each patient and then averaged across 30 Human Connectome Project datasets to obtain a patient-level mean absolute cosine similarity score.

Results:

Directional alignment between tumor progression vectors and local white matter fiber orientation was consistently observed when assessed using cosine similarity.

At the patient level, the mean absolute cosine similarity averaged across 30 Human Connectome Project datasets was 0.4167 ± 0.2710 (mean ± SD, $n = 29$), demonstrating substantial angular concordance.

This finding was consistent with the primary results obtained using the mean absolute Pearson correlation coefficient (MACC), supporting the robustness of the observed directional alignment with respect to the choice of sign-invariant alignment metric.
